# Fabrication of Biodegradable and Biocompatible Functional Polymers for Anti-Infection and Augmenting Wound Repair

**DOI:** 10.3390/polym15010120

**Published:** 2022-12-28

**Authors:** Shuhua Deng, Anfu Chen, Weijia Chen, Jindi Lai, Yameng Pei, Jiahua Wen, Can Yang, Jiajun Luo, Jingjing Zhang, Caihong Lei, Swastina Nath Varma, Chaozong Liu

**Affiliations:** 1 Guangdong Provincial Key Laboratory of Functional Soft Condensed Matter, School of Materials and Energy, Guangdong University of Technology, Guangzhou 510006, China; 2 Institute of Orthopaedics and Musculoskeletal Science, University College London, Royal National Orthopaedic Hospital, London HA4 4LP, UK; 3 Sino-German College of Intelligent Manufacturing, Shenzhen Technology University, Shenzhen 518118, China; 4 Centre for the Cellular Microenvironment, University of Glasgow, Glasgow G12 8LT, UK

**Keywords:** biodegradable, biocompatible, antibacterial, synthetic polymers, natural polymers, medical applications

## Abstract

The problem of bacteria-induced infections threatens the lives of many patients. Meanwhile, the misuse of antibiotics has led to a significant increase in bacterial resistance. There are two main ways to alleviate the issue: one is to introduce antimicrobial agents to medical devices to get local drug releasing and alleviating systemic toxicity and resistance, and the other is to develop new antimicrobial methods to kill bacteria. New antimicrobial methods include cationic polymers, metal ions, hydrophobic structures to prevent bacterial adhesion, photothermal sterilization, new biocides, etc. Biodegradable biocompatible synthetic polymers have been widely used in the medical field. They are often used in tissue engineering scaffolds as well as wound dressings, where bacterial infections in these medical devices can be serious or even fatal. However, such materials usually do not have inherent antimicrobial properties. They can be used as carriers for drug delivery or compounded with other antimicrobial materials to achieve antimicrobial effects. This review focuses on the antimicrobial behavior, preparation methods, and biocompatibility testing of biodegradable biocompatible synthetic polymers. Degradable biocompatible natural polymers with antimicrobial properties are also briefly described. Finally, the medical applications of these polymeric materials are presented.

## 1. Introduction

Infections, particularly implant-associated and hospital-acquired infections, present a global medical risk and cause serious injuries and deaths every year [1]. More than 23.5 million immunocompromised patients require immunosuppressive drugs, such as acquired immunodeficiency syndrome (AIDS), rheumatoid arthritis, and organ transplant patients. These drugs suppress the body’s normal immune response, making patients more susceptible to fungal and bacterial infections. For example, there are 300,000 HIV-related infections and 10,000 deaths in the United States every year [2]. In addition, exposure to other common pathogens such as *Pseudomonas aeruginosa* (*P. aeruginosa*), *Escherichia coli* (*E. coli*), *Salmonella*, and *Staphylococcus aureus* (*S. aureus*) can escalate into threatening infections that may lead to sepsis-related death [3]. It has been shown that the implant-associated infections are caused by incomplete preoperative sterilization procedures, the use of non-standard protocols during surgery, and hematogenous sources to the implant surface after surgery, resulting in financial hardship or even death for patients [4,5]. Infections caused by implants are usually attributed to bacteria, and the biofilm facilitates the bacteria adhering to the implant from the host’s defense system and bactericidal agents [6].

Further, opportunistic pathogen infections will pose a serious threat in organ transplants, prosthetic grafts, and tissue-engineered structures [7]. To solve this problem, some researchers have added antibacterial substances, such as silver and antibiotics, to the scaffold [8]. The overuse of antibiotics has led to the emergence of drug-resistant strains and a growing problem of bacterial resistance [9]. The scaffold may also lose its antimicrobial activity after the complete release of the antimicrobial substances [8]. New antimicrobial treatments are therefore essential for the control of hospital-acquired infections arising as a complication of surgery and post-surgery [10].

To alleviate the problems caused by antibiotic resistance, efforts have been made to develop polymer materials with antimicrobial properties featuring the ability to prevent bacteria from adhering or even to disrupt bacterial cell membranes [11]. In addition, the structure and shape of synthetic polymers can be engineered to satisfy medical requirements [12]. Amongst these, biodegradable and biocompatible polymers have attracted a great deal of attention. Typical antibacterial polymers stand out in terms of their chemical stability. Moreover, the polymer composition needs to be biocompatible and degradable in vivo and in vitro [13]. The biodegradability of polymers can be achieved by the presence or incorporation of unstable chemical linkages (e.g., ester, amide, and carbonate bonds) [14]. Biodegradable polymers are usually divided into two categories: synthetic polymers and natural polymers [15]. Biocompatible synthetic polymers are often biodegradable, and the products of degradation can be absorbed by the human body. Natural polymers are metabolized into metabolites that are easily cleared by the kidneys or can be used by the body [16]. Common synthetic polymers include polyglycolic acid (PGA), polylactic acid (PLA), poly(lactide-co-glycolide) (PLGA) and polycaprolactone (PCL). Some natural polymers, such as cellulose, gelatin, and chitosan, are also common degradable biocompatible polymers [17].

A variety of antimicrobial methods have been developed on degradable biocompatible polymers. For example, as an environmentally friendly class of antimicrobial agents, the cationic polymer is less likely to cause resistance because it kills bacteria primarily by disrupting cell membranes. Currently, cationic polymerization has been used in polymers such as waterborne polyurethane [18,19], and natural cationic polymers such as chitosan have been extensively studied for antibacterial purposes [20]. In addition, metal ions are also common additions to polymers for antibacterial purposes, such as Ag^+^, Zn^+^, Cu^+^, and so on. Degradable polymers achieve antimicrobial effect by slowly releasing metal ions. Nonionic antimicrobial polymers have also been extensively studied recently. Instead of interacting with bacteria through ions, nonionic polymers produce antimicrobial effects by adding some nonionic substances, such as curcumin, piperine, indole, aspirin, and so on [21]. These non-ionic antimicrobial agents may interact with bacteria due to hydrogen bonding, hydrophobic, dipole, etc. Although many non-antibiotics using antimicrobial methods have been investigated, the use of antibiotics is still a convenient and effective method for antimicrobial resistance. Degradable polymers not only allow the slow local release of antibiotics, but also reduce bacterial resistance compared to systemic antibiotic use. Therefore, the controlled release of drugs on degradable polymers is also a widely investigated topic.

For chronic osteomyelitis [22], bone tissue engineering [23], and wound healing [24], long-term antimicrobial therapy or avoidance of bacterial infection at the injury site is required. However, the long-term systemic use of antibiotics is very expensive and often causes systemic adverse effects [25]. Therefore, the focus of antibiotic therapy is to achieve high concentrations of antibiotics at the site of infection using a local delivery system to avoid the side effects associated with systemic administration. If a non-degradable drug delivery system is used, a secondary procedure is required for removal [26]. Therefore, efforts have been made to develop drug-delivery systems based on biodegradable biopolymers. The rate of drug release can also be regulated by adjusting the degradation rate of the biodegradable polymer.

This paper aims to review the antimicrobial behaviors and applications of biodegradable and biocompatible polymers in novel antimicrobial approaches developed for the alleviation of antibiotic resistance. These polymers hold great promise for applications, such as tissue engineering, drug delivery, and wound healing.

## 2. Approaches to Enhance Antibacterial Potential

**Figure 1:**
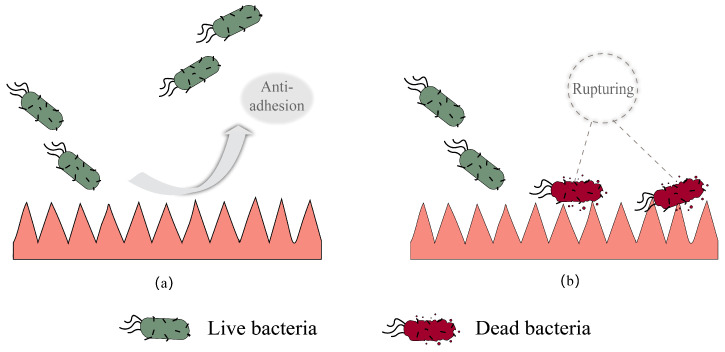
(**a**) Prevention of bacterial adhesion on microstructured surface; (**b**) destruction of bacterial cell membrane by surface microstructure.

Typically, two main methods are used to resist bacterial infection; the first method is the construction of antifouling biocompatible surfaces through hydrophilic polymers or superhydrophobic structures for resistance to bacterial adhesion [27]. Hydrophobic structures also can be inserted into the cell membrane, causing the rupturing of cell membrane, releasing cytoplasmic components (such as DNA and RNA), leading to the death of bacteria. Figure 1 shows the prevention of bacterial adhesion on a microstructured surface and the destruction of the bacterial cell membrane by surface microstructure. However, once bacteria are attached to the polymer surfaces, it will be difficult to inactivate the bacteria due to the formation of biofilms [28].

Yi et al. [29] developed a way to sterilize ZnO nanorods. The dead bacteria attached to the nanorods can be removed in an aqueous solution, thus restoring the antimicrobial properties of the microstructure. Wang et al. [30] prepared Zn-liganded polydopamine coatings that have surface microstructures and are superhydrophilic. Fajstavr et al. [31] used a high-energy excimer laser to treat the polydimethylsiloxane (PDMS) surface and obtained ripple-like surface nanopatterns. The nanopattern had a significant bactericidal effect. Li et al. [32] synthesized structurally homogeneous small-diameter mesoporous nanospheres that can convert hydrogen peroxide (H_2_O_2_) into reactive oxygen species for bactericidal effects. Cheng et al. [33] proposed activatable nanostructures. The structure provides an acoustodynamic sterilization method and enhances the proliferation and differentiation of osteoblasts. Nanostructures often produce antimicrobial effects through acoustodynamic or photodynamic catalysis or kill bacteria by physical puncture. Nanostructures can also be combined with other antimicrobial agents to produce a synergistic antimicrobial effect.

The second method uses antibiotics to actively kill bacteria and has a high bactericidal efficiency [27]. Antibiotics are a great invention. Since the discovery of penicillin, antibiotics have saved countless lives [34]. However, with the misuse of antibiotics, bacterial resistance has caused increasingly serious problems [35]. Despite ongoing efforts to find new drugs or alternatives to antibiotics, no antibiotic or its alternative have been clinically approved in the past 30 years [36]. To address the drawbacks of these two methods, some antimicrobial substances can be added to the hydrophobic surface to enhance the antimicrobial activity. Spasova et al. [37] prepared the superhydrophobic nanofiber materials of polyvinylidene fluoride and polyvinylidene fluoride-co-hexafluoropropylene by electrospinning technique and incorporated with ZnO nanoparticles. The addition of ZnO not only increased the surface hydrophobicity, but also imparted antibacterial properties to the materials. Mirzadeh et al. [38] prepared PU/TiO_2_ nanoparticles/graphene nanosheet composite films by the spraying technique. Non-solvent-induced phase separation increased the surface roughness and hydrophobicity of the films. The TiO_2_ was incorporated as an antimicrobial agent. Antibiotics can also be added to biodegradable materials to achieve local drug delivery and reduce systemic toxicity as well as drug resistance. Antibiotic-containing degradable polymer nanosystems (e.g., polymer vesicles and micelles) are used to deliver antibiotics [13]. One or more antibiotics are loaded into biodegradable polymer scaffolds for implantation into the body to accomplish the local release of antibiotics [39]. Studies have shown that bacterial cells carry more negative charges than mammalian cells do [40]. Therefore, this feature can also be exploited to explore new antibacterial methods, which can be bactericidal without affecting the normal growth of the cells. Cationic polymeric antimicrobial agents, such as polyhexamethylene biguanide and chitosan take advantage of this property. The positively charged cationic polymers rely on electrostatic attraction to bacterial cell membranes, which leads to cell membrane cleavage and bacterial death. Metal ions are also common bactericides, taking Ag^+^ as an example. The Ag^+^ can rely on electrostatic adsorption on the cell membrane, and peptidoglycan reaction with the cell wall, further penetrating the cell, leading to cell membrane cleavage. The Ag^+^ will react with enzymes and proteins after entering the cell, leading to the loss of normal cell function. The Ag^+^ also produces reactive oxygen radicals, ROSs, which bind to DNA and inhibit DNA, RNA, protein synthesis, thus leading to bacterial inactivation.

## 3. Synthetic Biodegradable Polymers for Antibacterial Applications

Synthetic biodegradable polymers are considered biocompatible and highly safe and have found numerous applications in the biomedical field [41]. Due to the degradable nature of polymer implants, they can be removed at the end of the functional life of the implant without surgical intervention. In tissue engineering, synthetic biodegradable polymers usually provide suitable mechanical properties and facilitate cell proliferation and differentiation, making it an excellent material for scaffolds [42]. Synthetic polymers have the advantage of being easy to process and mold, so there are more possibilities in terms of usage properties. Synthetic biodegradable polymers usually have no inherent antimicrobial ability. Therefore, the antimicrobial activity of synthetic polymers can be effectively improved by mixing with antimicrobial substances. Some inorganic substances can also be incorporated to endow them with antimicrobial ability. Different synthetic polymers have their own characteristics, such as degradation rate and hardness. To maximize their advantages, different polymers are often added to the material system to form composites or blends in previous studies. At the same time, these polymers have great potential as drug carriers, due in large part to their biodegradability, biocompatibility and the possibility of developing sustained/controlled/pulsed release and targeted drug delivery [43].

### 3.1. Polylactic Acid (PLA)

The PLA is one of the most widely used polymer materials for polymeric scaffolds in tissue engineering application because it has natural advantages of good mechanical properties, degradability, biocompatibility, and low cost [44]. However, the PLA has some limitations. For example, its hydrophobicity may undermine its biocompatibility. Human cells may be damaged if they are exposed to lactic acid, a degradation product of the PLA, for long periods of time [45]. Different parameters may result in the PLA exhibiting different mechanical properties, such as crystallinity, molecular weight, and processing [46]. The properties of the PLA can also be changed by tuning the material formulation, such as adding plasticizers, preparing blends, or composites with other materials. Thus, some chemical constituents are normally added into the PLA to improve various properties [47].

Llorens et al. [48] prepared the PLA nanofibers equipped with polybiguanide (PHMB) and with an average diameter between 560 and 630 nm by using the electrostatic spinning method. The PHMB-loaded PLA scaffolds have antimicrobial properties. On the one hand, the loading of PHMB increases the hydrophobicity of the scaffold, making it difficult for bacteria to adhere. On the other hand, the PHMB, as a cationic oligomer, has strong antibacterial activity. Scaffolds prepared by the electrostatic spinning method have porous structures that facilitate cell adhesion and proliferation. However, high concentrations of the PHMB are toxic to human cells, so it is important to control the concentration of the drug in the scaffolds and the rate of scaffold degradation. The experimental results showed that the concentration of PHMB did not affect the growth of cells when it was lower than 1.5 wt%. The PHMB-loaded PLA scaffolds showed biocompatibility in terms of the adhesion and proliferation of fibroblast and epithelial cell lines. Moreover, the slow and controlled drug release allowed the addition of PHMB in the scaffold at higher than safe concentrations.

Han et al. [45] proposed an innovative method for the preparation of PLA/chitosan (CS) composite films by using non-solvent induce phase separation (NIPS). The PLA/CS films prepared using the NIPS are more hydrophilic than those prepared using the casting method, which will result in better biocompatibility and a faster degradation rate of the films. The films prepared using the NIPS can also adjust the pore size of the porous structure to regulate the degradation rate. The PLA-based films were tested for their antibacterial activity against *E. coli*. The results showed that the antibacterial ability of the pure PLA film is not obvious due to the polymer structure of the PLA. The addition of CS greatly increases the antimicrobial activity, indicating that the CS is the main antimicrobial component in the film.

The porous film was experimentally demonstrated to be degradable, self-supporting, antibacterial, and transparent. The degradation of CS releases small alkaline molecules, which can neutralize the acidic degradation products of PLA and avoid some inflammation, resulting from acidic degradation to a certain extent.

Douglass et al. [49] combined the nitric oxide (NO) donor S-nitrosoglutathione (GSNO) with polyhydroxybutyrate (PHB) and the fiber-grade PLA for the manufacture of antimicrobial NO releasing nanofibers. In this study, the PLA was first mixed with the PHB in solution, stirred for 2 h, and then the GSNO was added into the solution. The fibers are formed by electrostatic spinning after 4 h. The fiber grade PLA synthesized with different length−diameter ratios overcomes some of its disadvantages, such as poor heat resistance and fragility. When the PLA was mixed with the PHB by a ratio of 3:1, the blends showed the best plasticity and maintained a certain tensile strength. The GSNO was added as a source of NO release because of the great role of NO in regulating antimicrobial behavior in blood vessels. Compared to PLA/PHB fibers, GSNO-containing fibers showed a significant reduction in colony forming units (CFU) measurements of *S. aureus* after both 2 and 24 h of exposure. After 2 h exposure, the bacterial survival rate of fibers containing GSNO was significantly lower than that of PLA/PHB fibers. The bacterial cell membrane on PLA/PHB remains intact, while that on PLA/PHB + 20 wt% GSNO is disrupted. This is due to the release of NO that disrupts the cell membrane.

Although the release of NO helps to kill bacteria, high concentrations of NO will be toxic to mammalian cells. Therefore, mouse fibroblasts were exposed to a 24 h leachate of fibers. The result shows that none of the fibers caused a significant decrease in cell viability compared to cells that were not exposed to the leachate. This demonstrates that nanofibers are not cytotoxic to mammalian cells. Therefore, NO-releasing nanofibers are a promising coating for blood-contacting medical devices.

Sharif et al. [50] synthesized composite scaffolds of the PLA/PCL blended with nano-hydroxyapatite (n(HA)) and cefixime-β cyclodextrin (Cfx-βCD) by electrospinning. In this study, the PLA and the PCL were mixed with the HA, Cfx, and Cfx-βCD to form composites film by electrospinning. Their antibacterial ability and effects on cell growth were compared. The antibacterial activity of the membrane was determined by using the *S. aureus* strain. After adding Cfx, the number of bacteria on the membrane decreased significantly within 24 h. The number of bacteria on the membrane continued to decrease when HA was added to the composite membrane. The best antibacterial activity was PLA-PCL-βCD-Cfx (PPH-βCD-Cfx) composite membrane.

The mouse pre-osteblast cell line (MC3T3) was cultured on the membrane to evaluate the cell viability on different membranes. The MC3T3 can attach and proliferate on all three membranes.

The addition of HA improved the osteoconduction of the composite. The βCD realizes the control of drug release as a carrier of antibiotics. The membranes were shown to have good antibacterial properties and to promote cell proliferation and attachment.

### 3.2. Polycaprolactone (PCL)

The PCL is a medical synthetic polymer with biocompatible and biodegradable properties. Due to its flexibility, low density, and easy processing, it has been widely used in tissue engineering scaffolds. In addition, it has good mechanical strength, rigidity, and heat resistance [51]. Its breakdown products form naturally occurring metabolites, which are readily metabolized by the body and eliminated without toxicity [52].

In previous research study, natural antimicrobial compounds, such as curcumin, piperine, eugenol, and rutin, were loaded into electrospun nanofibers based on the PCL [53]. A wound dressing was prepared by electrospinning. The SEM images showed that the nanofibers prepared by electrospinning were more uniform after adding curcumin and piperine. The diameter of the fiber can vary with the change in curcumin concentration. Novel three-component systems of curcumin–piperonin–eugenol (PCPiEu) and curcumin–piperonin–rutin (PCPiR) were designed and prepared. The growth of *S. aureus* in the presence of different wound dressings was studied. The growth of bacteria on pure PCL is tremendous, and so pure PCL has no antibacterial activity. With either the addition of curcumin or piperine, the number of bacteria was greatly reduced. In addition, the PC has almost no antibacterial activity against Enterococcus faecalis (Gram-negative), while both PCPiEu and PCPiR have a killing rate of more than 95%.

The MTT test was performed on different wound dressings using human fibroblasts to evaluate the cytotoxicity of wound dressings. The experiment proved that the pure PCL exhibited the highest amount of cell proliferation, which proves that the PCL has high biocompatibility. The result shows that the cell viability of piperine based on PCL samples is the lowest, which proves that piperine has cytotoxicity. The cell viability of PCR samples was higher than 100%, indicating that it was favorable for cell growth. In the three-component samples, the cell viability of PCPiEu and PCPiR was more than 90%. It seems that a better effect can be achieved by adjusting the amount of these natural compounds in the sample.

In general, the three-component wound dressing obtained good results in both the antibacterial test and the cytotoxicity test. In particular, it showed good antibacterial activity against Gram-negative bacteria. Although the mechanical properties of the system decreased compared with the PCL, the three-component wound dressing showed broad-spectrum antibacterial activity.

In another study, graphene (GP), bioglass, and zinc-doped bioglass were added to the PCL filaments, and their antimicrobial activity was analyzed comparatively. Materials for research were produced using the 3D-printing technique. The experimental results showed that the addition of a small amount of GP (0.5%) to PCL filaments resulted in a significant increase in antimicrobial activity compared to the pure PCL. This is because the structure of the GP may cause damage to the cell membranes of microbes and thus eliminate them [54].

Recently, PCL nanofibers containing Atropa belladonna were fabricated using the electrospinning technique [55]. The fruits, roots, and stems of belladonna are used in the treatment of many diseases and have strong antioxidant and anticancer properties. In this study, Atropa belladonna extract was used to encapsulate Ag nanoparticles (AgNPs).

This study concludes that both AgNPs and eAgNPs improved the antibacterial activity of PCL nanofibers against Gram-negative and Gram-positive bacteria. In addition, the cell viability of the PCL doped with eAgNPs was higher compared to the neat PCL. The main reasons for the greater cell viability of the PCL doped with eAgNPs may be that it is more hydrophilic than the pure PCL, the toxicity of AgNPs is reduced by coating on the surface of the nanoparticles, and the positive effect of free radicals in the Atropa belladonna structure on cell proliferation.

Felice et al. [56] synthesized PCL scaffolds compounded with hydroxyapatite (HA) and different concentrations of zinc oxide (ZnO) for bone tissue engineering by electrospinning techniques. The ZnO is an inorganic material with osteoinductive and osteoconductive properties. It has been reported that Zn^2+^ can induce osteoblast differentiation. In addition, ZnO is considered an effective antimicrobial agent against broad-spectrum microorganisms. The addition of HA is beneficial to increase the osteoconductivity of the scaffold and can shorten the degradation time of the PCL. To assess the antimicrobial activity of the scaffolds, a series of samples were immersed in the PBS at 37 °C for 0 and 30 days for in vitro degradation, respectively. Sterile samples were immersed in broth containing *S. aureus* for 18 h at 35 ± 2 °C, and the surviving CFUs were counted. The surviving CFU of samples after 0 and 30 days of degradation were compared after 18 h of bacterial incubation, respectively. The results showed that the presence of ZnO in the samples degraded for 0 days led to a reduction in the initial bacterial load compared with the negative control group. The higher the concentration of ZnO, the higher the antibacterial activity. On the PCL scaffold containing 6% ZnO, this corresponds to a 96% reduction in bacteria. On the PCL scaffold containing the HA and 1% ZnO, an almost 99% reduction of the initial bacterial load was achieved. Notably, on the scaffold containing the HA, the antibacterial activity decreased instead with increasing the ZnO concentration.

Human fetal osteoblast cell line (HFOb) proliferation was assessed after 3, 7, and 14 days of incubation on PCL, PCL:HA, and PCL:HA:ZnO nanofiber scaffolds. On the PCL and PCL:HA:ZnO 1%, cell proliferation increased exponentially with time. In contrast, cell proliferation in the other samples was constant from day 3 or from day 7. In addition, higher concentrations of ZnO may lead to reduced cell proliferation.

These scaffolds have been shown to have an antibacterial effect against *S. aureus*. This activity rises with increasing levels of the ZnO. However, high concentrations of the ZnO may reduce cell proliferation. Therefore, low-concentration ZnO scaffolds may be promising regenerative medicine products with antibacterial ability.

### 3.3. Polyglycolic Acid (PGA)

The PGA is a semi-crystalline synthetic polymer with good biocompatibility and biodegradability. Once it degrades, the non-crystalline part first will degrade to glycolic acid which can be readily metabolized by the body; the crystalline part then will degrade to harmless water and carbon dioxide [57]. Like the PLA, it produces acidic degradation products that may trigger inflammation. The PGA has high mechanical strength and high crystallinity. However, the PGA has poor toughness and a relatively high price. Therefore, the PGA is always blended with other polymer materials.

Shuai et al. [58] prepared polymer scaffolds by laser sintering. The PGA solution and the PLLA solution were mixed, stirred, and ultrasonically dispersed. Meanwhile, the graphene oxide (GO) solution and the nano Ag solution were mixed, stirred, and ultrasonically dispersed. Finally, the two solutions were mixed together, filtered, and dried to obtain powders. The powders were sintered layer by layer to form 3D scaffolds. The SEM images show that AgNPs and GO are evenly distributed. Both the GO and nano Ag are easy to form agglomeration, so uniform dispersion is very important for the antibacterial activity of polymer scaffolds. The *E. coli* suspension was placed together with scaffolds with different GO and Ag ratios for 24 h, and the antibacterial activity of the scaffolds was evaluated by turbidimetry. Only when GO exists, the antibacterial effect is not obvious. The antibacterial effect was significantly improved only when Ag was present. When GO and Ag were simultaneously present, the antibacterial effect was further increased.

**Figure 2:**
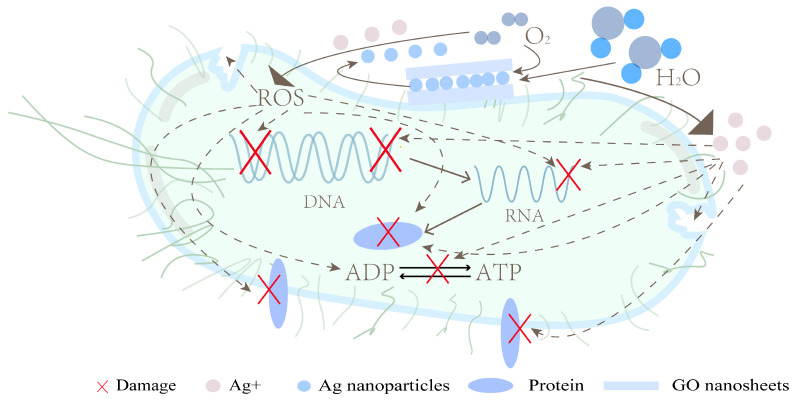
Synergistic antibacterial mechanism of GO-Ag.

The GO-Ag nanosystem showed a synergistic antibacterial effect by combining the trapping effect of GO nanosheets with the killing effect of Ag. The GO can interact with bacterial cell membranes and adsorb on bacterial cells, resulting in an increased concentration of AgNPs around the bacteria. The antibacterial effect of AgNPs mainly depends on the release of Ag^+^ and the promotion of reactive oxygen species (ROS) production. Figure 2 shows the synergistic antibacterial mechanism of the GO-Ag.

Among them, the scaffolds containing 1 wt% GO and 1 wt% Ag showed good cytocompatibility without affecting MG63 cell adhesion, viability, and proliferation because the presence of the GO has a positive effect on cell adhesion, and excessive Ag has a negative effect.

Wu et al. [59] found that total alkaloids from Semen Strychnine (TASS) was loaded into polyetheretherketone (PEEK)/PGA composite scaffolds prepared by 3D printing technology to obtain long-lasting antibacterial activity. The PEEK and PGA were dissolved in ethanol solution at a mass ratio of 6:4, and then different levels of TASS were dissolved in the PEEK/PGA suspension. The TASS-PEEK/PGA suspension was stirred magnetically for 2 h, and then the suspension was dried at 50 °C until the precipitate was of constant weight. Finally, the precipitate was ground homogeneously, and the scaffolds were prepared by selective laser sintering technique. The TASS has antibacterial, anti-inflammatory, and analgesic properties. The relationship between the antimicrobial activity of the scaffolds and the TASS content was investigated. As the TASS content increases, the antibacterial activity hinders both *S. aureus* and *E. coli* from proliferating.

However, the effective TASS level is close to the toxic dose, so making the TASS localized and controllable in release is critical. The human fetal osteoblastic cell line (hFOB 1.19 cells) was used to assess the cytotoxicity of the scaffolds. Different concentrations of TASS-PEEK/PGA were incubated with hFOB 1.19 cells for 1, 3, and 5 days. Compared to PEEK/PGA, 2.5% TASS-PEEK/PGA had a slight effect on cell survival. The 7.5% TASS-PEEK/PGA had fewer cells on the third day of incubation than on the first day, but the number of cells increased again on the fifth day. This may be due to faster drug release and higher TASS levels in the first three days. However, the slow release of the drug after that promoted the cell growth.

Gao et al. [60] reported a hyperbranched poly(amide-amine)-capped Ag shell and Au core nanoparticle (Ag@Au NP)-embedded fiber membrane-structured PGA/PLGA ureteral stent. This stent showed efficient sterilization properties, taking 5 min to kill *E. coli* and 10 min to kill *S. aureus*, respectively, with a 99% bacterial inhibition rate. In a 16-day in vitro assay, the stent showed durable antibacterial activity, low release of Ag and Au and low cytotoxicity. Gradient degradation of PGA/PLGA allowed constant exposure of Ag@AuNPs to the stent surface, which acted as a bactericide and eliminated bacterial adhesion.

### 3.4. Poly(Lactic-Co-Glycolic Acid) (PLGA)

The PLGA is a copolymer of the PLA and the PGA, which biodegrades faster than the PLA due to the presence of the PGA [61]. However, the surface properties of PLGA are not ideal for cell growth [62]. PLGA has been approved by the US Food and Drug Administration (FDA) for use as an implant, and PLGA has excellent mechanical properties and degradability. Surface modification of PLGA scaffolds is therefore a promising approach, which would provide useful surface properties to the polymer without changing the native properties [63].

Jing et al. [64] prepared nanoparticles based on PLGA-CS conjugates and PLGA-alendronate (Alen) conjugates. The PLGA-CS combines the good biocompatibility and biodegradability of PLGA and CS, facilitating drug delivery. Alen, a potent anti-osteoporosis drug, was used as a model drug for modifying the PLGA in this experiment. The cytotoxicity of the nanoparticles was assessed by CCK-8 assay. The cell survival rate was higher than 95% when nanoparticles with different concentrations were placed together with MC3T3 cells for 24 h. This indicates that the nanoparticles have no significant cytotoxicity to MC3T3 cells. To assess the specific cellular uptake of nanoparticles, nanoparticles with Alen (NP4) or without Alen (NP5) were incubated with HDF cells and MC3T3 cells for 3 and 24 h, respectively. The results showed that the uptake of NP4 into MC3T3 cells was significantly greater than that of NP5 after 24 h. This demonstrated that the Alen-modified nanoparticles had specific uptake into MC3T3 cells. Although the antimicrobial activity of nanoparticles was not tested in the article, the addition of CS may give the nanoparticles some antimicrobial activity.

De Faria et al. [65] prepared PLGA and CS blend fibers by using electrostatic spinning method. The PLGA-CS mats were functionalized with GO-Ag through a chemical reaction between the carboxyl group of GO and the primary amine functional group on PLGA-CS fibers.

To evaluate the antibacterial activity of PLGA-CS after modification with GO-Ag, unmodified PLGA-CS was used as a control. The GO-Ag modified PLGA-CS and PLGA-CS were exposed to *E. coli*, *P. aeruginosa* (Gram-negative), and *S. aureus* (Gram-positive) for 3 h. The result showed that the antibacterial activity was greatly improved after GO-Ag modification. The inactivation of *E. coli* and *P. aeruginosa* reached more than 98%, while the inactivation of *S. aureus* was lower at 79.4 ± 6.1%. This may be due to the thicker peptidoglycan layer of Gram-positive bacteria, which played a protective role against *S. aureus* cells.

Azzazy et al. [66] designed PLGA nanoparticles with CS coating and loaded with harmala alkaloid-rich fraction (HARF) (H/CS/PLGA) by the emulsion–solvent evaporation method. HARF has been reported to increase collagen and fibroblasts in the microenvironment near the wound, which allows it to accelerate wound healing. The lactic acid produced by the degradation of PLGA accelerates reparative angiogenesis, while CS has antibacterial, bioadhesive, and hemostatic properties. To evaluate the cytotoxicity of these nanoparticles, human skin fibroblasts were treated with different concentrations of H/CS/PLGA NPs to test cell viability. The cell viability was higher than 85% for all concentrations and did not differ significantly from the untreated cells.

The antibacterial activities of free HARF, CS/PLGA NPs, and H/CS/PLGA NPs were evaluated as shown in Table 1. The H/CS/PLGA NPs showed the highest antibacterial activity against *S. aureus* and *E. coli*. The nanoparticles can decompose near the wound and release loaded HARF, in which the alkaloids bind to bacterial DNA to act as antibacterial agents.

### 3.5. Summary of Antimicrobial Strategies for Degradable Synthetic Polymers

Antimicrobial applications of biodegradable synthetic polymers and mammalian cells used for testing biocompatibility are shown in Table 2.

These material systems were tested for their antimicrobial efficacy and biocompatibility. In PLA/PHMB, the complete growth inhibition of both bacteria occurred when the PHMB concentration was above 1.5 wt% (bacterial growth was lower than 1% compared to the PLA control). In contrast, when the PHMB concentration was below 0.75 wt%, bacterial growth was not significantly inhibited, but bacterial growth on the scaffold was still lower than that of the control. For the *E. coli*, it was about 30% lower than the control, and for the *M. luteus*, it was about 20% lower than the control. The antibacterial effect of the system was demonstrated to be dependent on the loading concentration of PHMB. For fibroblast and epithelial cell adhesion, it was 275% and 175% when the PHMB concentration was 1.5 wt% compared to the control, while it decreased to 175% and 100% when PHMB was increased to 2.5 wt%. This indicates that the system has good biocompatibility in a range of concentrations. Overall, low concentrations of PHMB inhibited bacterial adhesion and colonization due to the controlled and sustained release of PHMB. In contrast, significant inhibition of bacterial growth requires a concentration of PHMB higher than 0.75 wt%. In PLA/CS, the average antibacterial rate increased from 84.90% to 99.77% when PLA:CS was increased from 8:1 to 3:1. This indicates that the antimicrobial effect of the system depends on the relative content of CS. In the PLA/GSNO/PHB system, the bacterial adhesion rate of *S. aureus* was about 72.9% and 79.7% after 2 and 24 h exposure to the fiber. Only about 20% of the bacteria remained viable after 2 h of exposure. Mouse fibroblasts showed greater than 90% viability compared to the control group without fiber exposure. In PLA/PCL/n(HA)/cfx-βCD, the growth of *S. aureus* was reduced by 90% within 24 h. The cell viability of MC3T3 cells increased continuously from day 3 to day 7 and surpassed that of the control at day 14, contributing to cell proliferation. Antibacterial testing of *S. aureus* in PCL/curcumin/piperine/eugenol/rutin showed that the dressing containing piperine killed 100% of the bacteria and the dressing containing curcumin killed more than 90% of the bacteria. Two three-component systems, PCPiEu and PCPiR, achieved bactericidal rates of approximately 80%. Antibacterial tests on *E. faecalis* showed that curcumin had no antibacterial activity against this bacterium, while the three-component systems PCPiEu and PCPiR exhibited bactericidal rates of 99.47% and 96.88%. In cellular tests performed on human fibroblasts, the cell viability of the dressing containing only piperine was only 16.5%, whereas the three-component systems PCPiEu and PCPiR showed 94.2% and 98.5% cell viability. Therefore, the two three-component systems showed good overall performance in terms of antimicrobial and biocompatibility. In PCL/Atropa/AgNPs, the antimicrobial effect was brought about by AgNPs, and the antimicrobial effect was slightly reduced by the addition of Atropa. In contrast, in the cytotoxicity test of HaCaT cells, the cell survival rate was increased by about 30% with the addition of Atropa than with the addition of AgNPs only. In PCL/HA/ZnO, PCL/HA/ZnO 1% reduced the initial *S. aureus* bacterial load by almost 99%. In PGA/PLLA/GO/Ag, antimicrobial tests were conducted using *E. coli*. GO showed good synergistic bactericidal effect with Ag. GO itself had no significant bactericidal effect, while the addition of GO increased the bactericidal rate of PGA/PLLA/GO/Ag by 17.1% over PGA/PLLA/Ag to 95.4%. With the increase of Ag content to 1.5%, the bactericidal rate reached 99.9%. However, the 1.5% Ag content made the scaffolds less cytocompatible. While 1% content of Ag still maintained good biocompatibility. In PGA/PEEK/TASS, the antimicrobial rate of the scaffold reached 55.71% for E. coil and 15.84% for *S. aureus* when the TASS content was 7.5%. In the biocompatibility test, a TASS content of 2.5% better promoted the proliferation and differentiation of human fetal osteoblasts. Among PGA/PLGA/Ag@AuNPs, Ag@AuNPs showed strong bactericidal rates, reaching over 90% and 99.9999% against *E. coli* and *S. aureus*, respectively, and the bactericidal rate continued to increase with the increase of nanoparticles. The degradation of the outer layer of the scaffold does not release large amounts of Au and AgNPs, so the scaffold has good biocompatibility. In PLGA/CS/GO/Ag, the killing rate of both *E. coli* and *P. aeru-ginosa* was over 98%, while the inactivation rate of *S. aureus* was only about 79.4%.

It seems that all these antimicrobial methods have good bactericidal effect; however, they still have some drawbacks. For example, good bactericidal methods are often accompanied by strong cytotoxicity, which requires control of its concentration and release rate. Most of the methods are effective against only one type of bacteria but are not effective against others. More antimicrobial material systems need to be investigated in order to achieve an antimicrobial effect while promoting cell proliferation.

## 4. Natural Polymers

Natural polymers are derived from plant and animal organisms, and they are usually biocompatible, biodegradable, and non-toxic [67]. Materials of natural or biological origin are the first biodegradable biomaterials used in clinical practice [68]. The application of naturally derived polymers as bionics is attractive, as it includes motifs that are recognized by cells that facilitate cell adhesion and proliferation [69]. However, these materials usually have poor mechanical properties and are difficult to process. Therefore, chemical modifications are required to be used as suitable biomedical tools [70].

### 4.1. Chitosan

Chitosan is a polysaccharide mainly derived from the exoskeleton of crustaceans and has been developed to have a variety of therapeutic functions [71]. Chitosan has been shown to have higher antimicrobial activity, higher bactericidal rates, a wider range of activity, and low toxicity to mammalian cells [72,73]. The properties of chitosan can be modified due to the presence of reactive functional groups. Moreover, chitosan derivatives can be specifically produced to enhance the desired properties while maintaining their biocompatibility and degradation properties [74,75].

**Figure 3:**
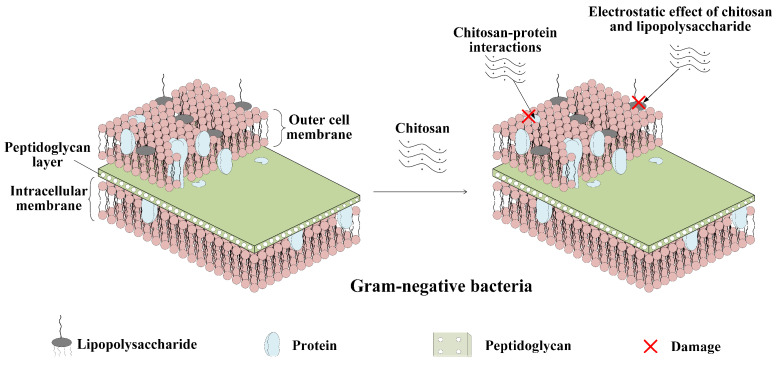
Bactericidal mechanism of chitosan against Gram-negative bacteria.

The chitosan has antibacterial activity against many bacteria and fungi, and this unique property is mainly attributed to the polycationic nature of chitosan [76]. The antibacterial effect of fungal chitosan against the Gram-negative bacterium *E. coli* can be strongly attributed to its interaction with cell membrane components, as evidenced by the dramatic morphological changes in cell shape and structure following chitosan treatment [77]. Gram-negative bacteria usually have complex cell walls [78], and the antimicrobial effect of chitosan may be due to the biochemical attachment caused by the cationic charge on the chitosan particles and the anionic charge of the cell wall components [79]. In addition, proteins covalently linked to peptidoglycan in the cell membrane are largely responsible for the antigenic properties of bacteria and can interact strongly with charged chitosan particles, leading to cell death [77]. Figure 3 shows the bactericidal mechanism of chitosan against Gram-negative bacteria.

**Figure 4:**
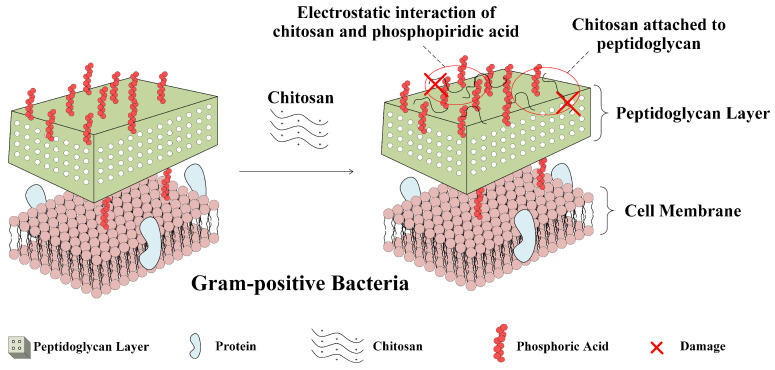
The antibacterial mechanism of chitosan against Gram-positive bacteria.

Gram-positive bacteria usually have a thick peptidoglycan layer. The chitosan has a high ability to attach peptidoglycans as an acidic polymer [80]. In addition, the phosphopeptidic acid on peptidoglycan has a polyanionic nature, and the electrostatic interaction between chitosan and phosphopeptidic acid disrupts the function of phosphopeptidic acid and causes functional disorders in bacteria. Figure 4 shows the antibacterial mechanism of chitosan against Gram-positive bacteria.

Chitosan is widely studied in medical applications because of its unique antibacterial properties and good biocompatibility. Chen et al. [81] prepared the polyelectrolyte components (PEC) with good biocompatibility by polyelectrolyte assembly, using carboxymethyl starch (CMS) and chitosan oligosaccharides (COS). The CMS/COS-PEC has controlled physicochemical properties and antibacterial activity against *S. aureus*. It can be used as a degradable hemostatic agent. Hu et al. [82] reported for the first time an innovative three-dimensional porous chitosan-vanillin-bioglass (CVB) scaffold with vanillin and bioglass as non-toxic and osteoconductive cross-linkers. The scaffold had strong antibacterial activity and improved mechanical properties. With high porosity, it significantly promoted osteoblast differentiation.

### 4.2. Gelatin

Gelatin is a biopolymer easily obtained from the partial hydrolysis of collagen and is one of the most used biopolymers approved by the FDA due to its biocompatibility, biodegradability, and ease of access [83]. In addition, the process of gelatin degradation produces little inflammatory response [84]. The degradation products are amino acids, which benefit the body, support cell adhesion, and promote cell proliferation [85,86].

Polyurethane/gelatin hybrid nanofiber scaffolds were prepared by using electrostatic spinning to promote cell growth and barrier to bacteria [86]. Cryogels cross-linked with gelatin and dopamine are effective hemostatic materials and can promote wound healing. Dopamine confers near-infrared radiation-assisted antimicrobial capacity to the cryogel [87]. Zhao et al. [85] also developed a gelatin-based physical double-network hydrogel with photothermal antibacterial activity. Photothermal sterilization holds promise as a physical antimicrobial method to kill multidrug-resistant bacteria. Metals and metal compounds, such as Au/Ag [88] and Zno [89], have also been added to gelatin to obtain antimicrobial activity.

### 4.3. Cellulose

Cellulose is a linear polysaccharide found in large quantities in several natural sources [90]. Cellulose and its derivatives are biocompatible polymers that have attracted considerable interest for biomedical applications due to their suitable physical and mechanical properties [91].

Orlando et al. [92] have suggested a green chemistry strategy to the functionalization of cellulose for the introduction of antibacterial functional groups. In the case of the functionalized material compared to the unmodified material, a reduction in the bacterial cell population by roughly half was seen within 24 h for both strains of *S. aureus* and *E. coli*. Additionally, cytotoxicity tests have shown that cellulose hydrogels do not harm keratin-forming cells when they come into touch with them directly or indirectly, even after exposure for 6 days. Fernandes et al. [93] obtained antimicrobial bacterial cellulose membranes by chemically grafting aminoalkyl groups onto the surface of their nanofiber network. These bacterial cellulose membranes showed antibacterial activity against both *S. aureus* and *E. coli*, whereas they were non-toxic to human-adipose-derived mesenchymal stem cells. Hassanpour et al. [94] chemically modified the surface of nanocellulose with a phenanthrene silica salt. The modified samples showed promising results against both Gram-positive (*S. aureus*) and Gram-negative (*E. coli*) bacteria. Moreover, it does not affect the viability of normal HDF cells at low concentrations.

The antimicrobial strategies and biocompatibility tests based on natural polymers are shown in Table 3.

## 5. Application of Biodegradable Biocompatible Polymers

### 5.1. Wound Healing

Wound healing is a natural physiological process that occurs in response to any tissue injury or damage [95]. There is a high risk of infection during the healing process because damaged skin cannot form a proper barrier to bacteria. Therefore, antibiotics are often used to combat bacteria and significantly reduce the mortality rate from bacterial diseases worldwide [96]. The use of antibiotics (e.g., penicillin, vancomycin, and gentamicin) is currently the standard in the management of bacterial infections [97]. Unfortunately, mutations in bacteria have led to a resistance to antibiotics for them, and it has significantly changed the trend toward treatment [98]. Therefore, an ideal wound dressing should have proper mechanical properties, excellent hemostatic properties, and air permeation as well as antibacterial activity [99,100].

Natural or synthetic polymers with biocompatible and biodegradable properties play a crucial role in cell adhesion and proliferation because of their unique structure and excellent mechanical properties [101,102]. For example, chitosan promotes wound healing by enhancing the migration of fibroblasts and the deposition of collagen in the wound area. In addition, chitosan has hemostatic and antibacterial properties [103,104,105]. The degradation product of PLGA and lactic acid accelerates the repair of blood vessels and facilitates wound healing.

### 5.2. Tissue Engineering

The intricate hierarchical structure of human tissues makes them susceptible to injury, cancer, and some degenerative diseases over the course of a person’s lifespan [106]. Therefore, the regeneration and repair of damaged tissues are very important for the healing process. Tissue engineering is the most promising approach, promoting cell growth by implanting biomaterials [107]. In bone tissue engineering, osteoblasts, chondrocytes, and mesenchymal stem cells are obtained from the patient’s hard and soft tissues. Then, they are propagated in cultures and inoculated onto the scaffold. The scaffold is implanted into the patient, slowly degrades, and resorbs as the tissue structures grow [108,109].

Orthopedic implants exhibit good biocompatibility, biodegradability, porosity, and mechanical strength, but lack antimicrobial ability [110]. The development of aseptic surgical techniques and prophylactic systemic antibiotic therapy have reduced the incidence of infection. However, the bacterial colonization of medical devices or implants is still a serious risk [111,112]. With the increase in antibiotic-resistant bacteria, new antimicrobial approaches are being explored [113]. Most of these methods are developed on degradable biocompatible polymers.

As we have known, gelatin mixes well with natural and synthetic polymers to promote high biomechanical and bioaffinity of the scaffold. The 3D porous scaffolds and nanofiber scaffolds prepared with gelatin as the main material are mainly used for large bone defects [114]. The PCL [115,116], PGA, PLA, and PLGA copolymers [117,118] are the most used synthetic biodegradable polymers for 3D scaffolds in tissue engineering. For example, many variants of the PCL facilitate the induction of bone tissue differentiation. The PLA and the PGA are easy to process, and their degradation rates and physical and mechanical properties can be adjusted over a wide range by changing parameters [119,120].

### 5.3. Drug Deliver

Conventional antimicrobial drugs face a few difficulties, including frequent resistance, formulation-related restrictions, subpar drug targeting, and subpar drug release, all of which can result in toxicity in mammalian cells or ineffectiveness at the site of action [121]. Global human health is plainly at risk due to the growing resistance to already prescribed antibiotics and the declining availability of novel antibiotic medications. Therefore, one of the main areas of attention for the internationally acknowledged research priority is the quest for new and efficient techniques to improve medication therapy against existing antibiotics [122].

To balance the biochemical events of inflammation in chronic wounds and promote healing, chitosan-based hydrogels are well-suited for intelligent administration and can be loaded with antibacterial agents, growth factors, stem cells, and peptides [123]. Gelatin is a flexible biopolymer that has historically made it possible to develop a variety of drug delivery methods, including fibers, hydrogels, microparticles, and nanoparticles. These various methods all have certain qualities that make them particularly well-suitable for medication delivery [124]. After being effectively used to entrain antimicrobial agents, the PCL is widely used as a drug delivery method to promote bone growth and regeneration in the treatment of bone diseases [125].

## 6. Future Prospects and Challenges

Injuries and deaths caused by infections have become a global public health concern. With the emergence of multiple drug-resistant strains, however, the problem of bacterial resistance has become increasingly serious. Therefore, there is an urgent need to develop new antimicrobial methods to combat the problems caused by infections. Biodegradable biocompatible polymers have become a hot topic of research, both as scaffolds for tissue engineering and as carriers for drug delivery. Biodegradable and biocompatible polymers can be divided into synthetic polymers and natural polymers. Synthetic polymers have good mechanical properties and stability but lack the ability to grow cells. Synthetic polymers often have no antimicrobial activity. Therefore, antimicrobial agents are often added to synthetic polymers to endow them with antimicrobial activity. These antimicrobial agents include metals, antibiotics, and some antimicrobial agents derived from plants. However, all these methods have some shortcomings, such as the tendency of metal ions to cause toxicity to cells when killing bacteria. Long-term use of antibiotics may lead to drug resistance, yet new antibiotics are rarely invented. Plant-derived antimicrobial agents require strict dosage control. Otherwise, they may be toxic to cells instead of being effective. Therefore, modulating the degradation rate of degradable polymers is a promising direction, which could lead to the controlled release of antimicrobial agents. Natural polymers have good biocompatibility, but rather poor mechanical properties and are not easy to process. Several methods have been reported to improve their disadvantages, and antimicrobial features have been developed on natural polymers. Natural polymers have a variety of origins and can be chemically modified to obtain several variants. Due to their poor mechanical properties, they are mostly used to fabricate wound dressings or hydrogels. There are also many studies on blending natural polymers with synthetic polymers to exploit their respective advantages. Some physical sterilization methods have also been investigated, such as the construction of surface microstructures to puncture bacterial biofilms and near-infrared light irradiation for sterilization. Therefore, biodegradable biocompatible polymers with antimicrobial properties and the ability to promote cell growth will be a promising approach to alleviate the problem of antibiotic resistance.

Degradable polymers play a key role in the sustained release of drugs and more and more antimicrobial methods are being developed on degradable polymers. For antimicrobial substances incorporated into degradable polymers, however, the antimicrobial activity is often accompanied by cytotoxicity more or less. Therefore, controlling the sustained, stable, and quantitative release of drugs is still a subject that needs to be investigated in depth.

## 7. Conclusions

Infections caused by bacteria can be very dangerous to people’s health. The most effective way to traditionally fight back against bacteria is the widespread use of antibiotics. However, due to the misuse of antibiotics, a variety of drug-resistant bacteria have emerged. Therefore, there is an urgent need to seek alternative antimicrobial methods. Biodegradable and biocompatible functional polymers are widely used in medical treatments as tissue engineering scaffolds and wound dressings. These medical devices are susceptible to bacterial infections. Moreover, a great deal of effort has been put into the research of biodegradable polymers with antimicrobial properties. Currently, effective antimicrobial methods include cationic polymer antimicrobial, metal ion antimicrobial, hydrophobic structure to prevent bacterial adhesion, photothermal sterilization, nonionic antimicrobial agents, etc. Meanwhile, biodegradable polymers can be used as carriers for drug delivery to load antibiotics, thus achieving local release of antibiotics. Biodegradable polymers with antibacterial functions have a wide range of applications, including the prevention of bacterial infections caused by medical devices and the construction of drug delivery systems.

## Figures and Tables

**Table 1:** Antibacterial activity of optimal H/CS/PLGA NPs and free HARF against *S. aureus* and *E. coli*.

Bacterial Strain	Minimum Inhibitory Concentration (MIC in mg/mL)		
	HARF	CS/PLGA NPs	H/CS/PLGA NPs
*S. aureus*	0.5	0.18	0.13
*E. coli*	0.5	0.18	0.06

**Table 2:** Summary of antimicrobial applications and biocompatibility testing of degradable synthetic polymers.

Systems	Approaches	Bacterial Strains	Mammalian Cells Used for Testing	Antibacterial Mechanism	Refs.
PLA/PHMB	Electrostatic spinning	*E. coli* *M. luteus*	fibroblast and epithelial cell lines	PHMB cationic polymer antibacterial	[48]
PLA/CS	Non-solvent induce phase separation	*E. coli*	-	CS cationic polymer antibacterial	[45]
PLA/GSNO/PHB	Electrostatic spinning	*S. aureus*	fibroblasts	GSNO releases NO to modulate the polarity shift of macrophages and produce anti-inflammatory effects.	[49]
PLA/PCL/n(HA)/cfx-βCD	Electrostatic spinning	*S. aureus*	Mouse pre-osteblast cell line	Antibacterial effects caused by antibiotics	[50]
PCL/curcumin/piperine/eugenol/rutin	Electrostatic spinning	*S. aureus* *Enterococcus faecalis*	fibroblasts	Antibacterial effect of natural plant extracts	[53]
PCL/GP/Bioglass	3D printing	*S. aureus* *E. coli* *C. albicans*	-	Structure of GP disrupts bacterial cell membrane	[54]
PCL/Atropa/AgNPs	Electrostatic spinning	*S. aureus* *E. coli*	HaCaT cells	The bactericidal effect of Ag^+^	[55]
PCL/HA/ZnO	Electrostatic spinning	*S. aureus*	Human fetal osteoblast cell line	Release of Zn^+^ from ZnO to produce antibacterial effect	[56]
PGA/PLLA/GO/Ag	Laser sintering	*E. coli*	MG63 cells	The trapping effect of GO is synergistically bactericidal with Ag^+^ bactericidal action.	[58]
PGA/PEEK/TASS	3D printing	*S. aureus* *E. coli*	Human fetal osteoblast cell line	Antibacterial effects caused by antibiotics	[59]
PGA/PLGA/Ag@Au NPs	-	*S. aureus* *E. coli*	L929 cells	Metal ion sterilization effect	[60]
PLGA/CS/Alen	-	*-*	Mouse pre-osteblast cell line	CS cationic polymer antibacterial	[64]
PLGA/CS/GO/Ag	Electrostatic spinning	*S. aureus* *E. coli* *P. aeruginosa*	-	1. CS cationic polymer antibacterial 2. The trapping effect of GO is synergistically bactericidal with Ag^+^ bactericidal action.	[65]
PLGA/CS/HARF	Emulsion-solvent evaporation	*S. aureus* *E. coli*	Human skin fibroblasts	1. CS cationic polymer antibacterial 2. Antibacterial effect of alkaloids	[66]

**Table 3:** Summary of antimicrobial applications and biocompatibility testing of degradable natural polymers.

Systems	Approaches	Bacterial Strains	Mammalian Cells Used for Testing	Antibacterial Mechanism	Refs.
CMS/COS	Polyelectrolyte assembly	*E. coli* *S. aureus*	MC3T3-L1 fibroblasts	Antibacterial activity of COS	[81]
Vanillin/bioglass/chitosan	Crosslinking	*S. gordonii* *S. sanguinis*	MC3T3-E1	Antimicrobial effect of chitosan	[82]
Modified gelatin/Fe^+^	Crosslinking	*E. coli* *S. aureus*	-	Near-infrared radiation photothermal antibacterial	[85]
Gelatin/polydopamine	Crosslinking	*E. coli* *S. aureus*	L929 cells	Near-infrared radiation photothermal antibacterial	[87]
Au/Ag@gelatin	-	*P. aeruginosa*	-	Produces ROS under white light irradiation, which kills bacteria.	[88]
Gelatin/ZnO	Electrostatic spinning	*S. aureus* *E. coli*	MRC-5 cells	ZnO generates superoxide radicals and damages bacterial cell walls	[89]
Chemical modification of bacterial cellulose	Crosslinking	*S. aureus* *E. coli*	HaCaT cells	Introduction of antimicrobial groups	[92]
Chemical modification of bacterial cellulose	Crosslinking	*S. aureus* *E. coli*	Human adipose-derived mesenchymal stem cells	Introduction of antimicrobial groups	[93]
Chemical modification of nanofibrillated cellulose	-	*S. aureus* *E. coli*	Human dermal fibroblasts	Introduction of antimicrobial groups	[94]

## Data Availability

Not applicable.
